# Dr. Henry E. Allison

**Published:** 1905-01

**Authors:** 


Dr. Henry E. Allison, medical superintendent of the Matteawan State Hospital, died at Newburg, N. Y., November 12, 1904, of Bright's disease, aged 53 years. He received his doctorate degree from Dartmouth Medical College in 1878, and served as assistant physician at Willard Asylum, Ovid, N. Y. In 1889 he became medical superintendent of the state asylum for insane criminals at Auburn, N. Y. He was appointed a member of the commission to found a new asylum for insane criminals at Matteawan and, on its completion in 1892, the inmates of the old Auburn asylum were transferred there. Dr. Allison became medical superintendent and treasurer of the new institution, which place he held until his death.

